# A comparative study on the credibility of ischemic stroke treatment information on social media platforms: evidence from Weibo and REDnote

**DOI:** 10.3389/fdgth.2026.1858554

**Published:** 2026-08-26

**Authors:** Jiayan Gu, Zihan Li, Jiajun Yang, Sen Miao, Juan Li, Lijuan Gu

**Affiliations:** 1 Central Laboratory, Renmin Hospital of Wuhan University, Wuhan, China; 2 Hubei Province Key Laboratory of Traditional Chinese Medicine Resource and Chemistry, College of Pharmacy, Hubei University of Chinese Medicine, Wuhan, China; 3 Hubei Shizhen Laboratory, Wuhan, China

**Keywords:** health information, ischemic stroke, large language models (LLMs), REDnote, Weibo

## Abstract

**Background:**

Social media is a key channel for public health information, but its open nature leads to mixed quality of information regarding Ischemic Stroke (IS) treatment, which may mislead patient decisions. This study aimed to systematically compare IS treatment information published by professional and lay sources on two major Chinese social media platforms, Weibo and REDnote, in terms of content, engagement, and quality.

**Methods:**

This study employed computational social science and Natural Language Processing (NLP) techniques to analyze posts from four sources (Weibo-Pro, Weibo-Lay, REDnote-Pro, REDnote-Lay). We used topic modeling and co-occurrence networks to analyze content features and developed an automated scoring system based on a Large Language Model (LLM) to quantitatively evaluate information quality on two dimensions: “linguistic features” and “evidence-based medical content”.

**Results:**

Lay-sourced content exceeded professional-sourced content in both volume and user engagement. Content and quality patterns differed across the two platforms: on Weibo, the evidence-based quality of professional content was significantly higher than that of lay content (*p* < 0.001), whereas no significant professional–lay difference was detected on REDnote. We refer to this observed convergence as a “quality paradox,” while recognizing that the cross-sectional design does not establish a platform effect. Content themes also differed: Weibo-Lay content centered on Traditional Chinese Medicine, whereas REDnote-Lay content focused on rehabilitation experiences and family support.

**Conclusion:**

Distinct platform- and source-related patterns were observed across four “discursive communities.” On REDnote, professional- and lay-sourced posts showed similar evidence-based quality in this sample. These findings support further investigation of platform-specific communication environments and may inform cautious, context-sensitive approaches for patients, clinicians, and platform managers.

## 1 Introduction

Ischemic stroke (IS), as a major cerebrovascular disease, has posed a serious challenge to public health in China. Epidemiological data show that IS is the leading cause of death among Chinese residents, with a particularly prominent disease burden globally (1). From 1990 to 2021, the incidence of IS in China exhibited a significant upward trend (2), imposing enormous economic and caregiving pressures on society and the healthcare system (3). Notably, the disease has shown a younger onset trend, posing a severe threat to the long-term health and productivity of middle-aged and young populations (2). In addressing the challenges of such chronic diseases, health information plays a crucial role. Timely, accurate, and easily understandable health information is essential for effective disease prevention, supporting treatment decisions, and promoting rehabilitation management (4). Studies have shown that high-quality health information can significantly enhance patients' health literacy and self-management capacity, thereby improving treatment adherence and ultimately health outcomes (5).

With the development of digital technology, social media has become an important channel for the public to obtain health information (6). In China, Weibo and REDnote (also known as Xiaohongshu) are two representative platforms with large user bases and distinct communicative affordances. Weibo reports approximately 588 million monthly active users, whereas REDnote has more than 330 million (7). These functional differences make both platforms worth considering because they represent complementary communication environments: Weibo captures open, authority-oriented health information dissemination, whereas REDnote captures visual, experience-based lay health communication embedded in everyday lifestyle narratives. On these platforms, health information producers present a distinct dual structure: one category is professional-sourced information released by medical institutions or certified professionals, while the other is lay-sourced information generated by ordinary users or patients sharing personal experiences. These two types of information sources differ fundamentally in content logic, dissemination mechanisms, and discourse systems, together forming a complex online health information ecosystem (8). Representative interfaces are provided in Supplementary Figure S3 to illustrate the empirical setting and platform affordances examined in this study.

Although social media has greatly improved the accessibility of health information, its openness and decentralized dissemination model also lead to mixed information quality (9). A large volume of unverified, misleading, and even harmful information coexists with authoritative knowledge, creating significant risks for public judgment and decision-making (10, 21). Misinformation may mislead patients into adopting ineffective or harmful treatment strategies, delaying optimal treatment opportunities. However, current academic research on the quality assessment of health information on social media remains limited. In particular, systematic and quantitative comparative studies focusing on IS, a high-burden disease, across different platforms (e.g., Weibo and REDnote) and different information sources (Professional-sourced vs. Lay-sourced) are lacking. This research gap prevents a comprehensive understanding of the strengths and weaknesses of different platforms and information sources in health communication and constrains the development of evidence-based intervention strategies.

To fill this research gap, the present study aims to conduct a multidimensional comparative analysis of IS treatment information on Weibo and REDnote. This gap is addressed by comparing professional- and lay-sourced IS treatment information across two platforms with distinct communicative affordances through three complementary dimensions: dissemination, content characteristics, and information quality. These dimensions allow us to assess not only how widely different types of information circulate, but also what they communicate and how reliable or useful they are for the public. To make these objectives more explicit, the analysis is guided by three interrelated research questions. First, we ask how the content volume and user engagement metrics differ across four types of information sources: Weibo-Pro, Weibo-Lay, REDnote-Pro, and REDnote-Lay. Second, we examine how the thematic and presentational characteristics vary across the four sources. Third, we assess how a multidimensional quantitative scoring system can be developed and applied to evaluate and compare content quality across the four sources, while exploring its potential for tool-based application.

The significance of this study lies in providing empirical evidence on the quality of social media health information for the public, clinicians, and platform managers. The findings may help users and clinicians recognize platform- and source-related differences and may inform future research on information review, recommendation, and communication strategies. Because the study is observational and did not evaluate interventions, these implications should be regarded as preliminary rather than prescriptive.

## 2 Research methods

This study adopted a multi-stage research methodology that integrates computational social science and natural language processing (NLP) techniques, aiming to systematically collect, process, analyze, and evaluate information related to IS treatment on two major social media platforms in China: Weibo and REDnote. All data processing and analyses were conducted in the R environment (version 4.3.2), with external large language model (LLM) APIs also utilized. The research was conducted in four sequential stages. First, publicly available posts were collected from Weibo and REDnote and then cleaned and preprocessed. Second, descriptive and content analyses were performed, including overlap, geospatial, thematic, conceptual, and viral-content analyses. Third, the quality of health information was quantitatively evaluated through a structured scoring procedure and reliability testing. Fourth, statistical analyses were conducted to compare differences across platforms and information-source types.

### 2.1 Data collection and preprocessing

Using Bazhuayu Collector 8, the study collected a large volume of publicly available posts on IS treatment from Weibo and REDnote, the data collection was conducted from July 31 to August 6, 2025, with July 31, 2025 set as the data cutoff date for eligible posts. The collection was based on a tailored keyword strategy. To distinguish between different information sources, two keyword lexicons were constructed: a “formal lexicon” (including “ischemic stroke therapy”, “ischemic stroke treatment”, “treating ischemic stroke”) and a “colloquial lexicon” (including “treat stroke (Zhong feng)”, “treat cerebral infarction”, “stroke treatment”, “cerebral infarction treatment”, “how to treat stroke”, “how to cure stroke”, “how to treat cerebral infarction”, “how to cure cerebral infarction”).

The raw data were then subjected to a rigorous cleaning and preprocessing pipeline. Posts with empty content were first removed. Next, the distinct() function from the “dplyr” package was used to deduplicate posts based on textual uniqueness, ensuring that each analyzed post was unique. Finally, the “lubridate” package was applied to standardize publication times into a consistent datetime format.

### 2.2 Descriptive and content analyses

After preprocessing, the study conducted descriptive and content analyses across multiple dimensions.

#### 2.2.1 Overlap and geospatial analyses

To explore content overlap across the four categories of data sources, the “ggvenn” package was used to generate Venn diagrams that visually illustrate the number of overlapping posts. For geospatial analysis, user-provided location data were extracted. The “sf” package was used to read a JSON map of China, while “ggplot2” and “scatterpie” were applied to visualize provincial-level data: background shading represents the total volume of relevant posts, while pie charts depict the proportion of content across the four categories within each province.

#### 2.2.2 Thematic and conceptual structure analyses: a two-stage approach

To comprehensively analyze the thematic structures within the four source categories, we employed a two-stage analysis strategy combining a traditional topic model for exploratory analysis with a contextualized model for in-depth validation, an approach that has been increasingly adopted in computational social science to balance interpretability and contextual sensitivity.

Stage 1: Exploratory Analysis using Latent Dirichlet Allocation (LDA). As an initial step, we utilized LDA to identify broad, high-level topics. The number of topics (K) was optimized by calculating the C_v coherence score across a range of values from 2 to 10, with *K* = 2 providing the most coherent and interpretable thematic structure (11). The model was implemented using the “topicmodels” R package with the standard Gibbs sampling method, and the Dirichlet hyperparameters were set to *α* = 0.1 and *β* = 0.1.

Stage 2: In-depth Analysis using “BERTopic”. To overcome the limitations of the bag-of-words assumption in LDA and to capture the contextual nuances of social media content, we conducted a second, more advanced analysis using “BERTopic”. This approach leverages the “paraphrase-multilingual-MiniLM-L12-v2” sentence-transformer model to generate semantically rich document embeddings. These embeddings were then processed through “UMAP” for dimensionality reduction and “HDBSCAN” for clustering to identify fine-grained topics. In the original BERTopic implementation, UMAP was run with the default settings: n_neighbors = 15, n_components = 5, min_dist = 0.0, metric = “cosine”, and low_memory = FALSE. HDBSCAN was configured with min_cluster_size = 15, metric = “euclidean”, cluster_selection_method = “eom”, and prediction_data = TRUE; min_samples was not manually specified and therefore followed the default setting. This two-stage approach allowed us to first identify overarching themes with LDA and then validate and refine them with the context-aware BERTopic model.

#### 2.2.3 Analysis of viral content characteristics

To compare topics associated with relatively high engagement while accounting for large differences in engagement distributions across platforms and source categories, posts were ranked by number of likes separately within each of the four platform-source categories. Within each category, posts at or above the 75th percentile were operationally classified as “viral” (high-engagement), and the remaining posts were classified as non-viral. The distributions of likes were highly right-skewed, so a single absolute cutoff would have disproportionately selected posts from groups with higher baseline engagement. The upper quartile provided a transparent relative contrast while retaining 25% of each category for subsequent modeling. This operational definition denotes relative high engagement within a category and should not be interpreted as an absolute or universally accepted definition of virality. A Structural Topic Model (STM) was then applied using the “stm” package, with “viral vs. non-viral” status set as a covariate (12). Forest plots were used to visualize differences in topic prevalence between high-engagement and other posts.

### 2.3 Quantitative evaluation of content quality

A central methodological component of this study is an automated, multidimensional content-quality scoring framework based on an LLM. The framework was designed to support transparent, reproducible, and scalable analysis; its performance was examined through test-retest reliability and preliminary comparison with consensus manual ratings. All interactions with the LLM were conducted via the API of the “doubao-seed-1-6-flash-250715” model, provided by Volcengine. The system consists of two scoring dimensions: “linguistic features” and “evidence-based medical content.”

#### 2.3.1 Scoring procedure

For linguistic feature scoring, the study invoked the “Doubao” LLM API through the “httr” and “jsonlite” packages (13). By means of carefully engineered prompts, the LLM was instructed to quantify aspects such as stance, objectivity, and logical coherence of the text. Briefly, the linguistic scoring prompt required the LLM to first identify the overall stance of each post, such as supportive, critical/refutational, or neutral reporting, and then assign a score according to predefined positive and negative indicators. Positive indicators included references to high-quality evidence, authoritative sources, scientific terminology, and cautious or objective wording, whereas negative indicators included reliance on personal anecdotes or hearsay, absolutist or miracle-cure claims, marketing language, and pseudoscientific expressions. The LLM was also required to provide a short reasoning statement to justify each score. The complete and unabridged prompt for linguistic feature scoring is provided in Supplementary Appendix S1.

For evidence-based medical content scoring, the process involved linking the LLM with external databases: first, the LLM extracted core treatment names from posts and translated them into English. The full prompt for this extraction task is available in Supplementary Appendix S2; next, the “rentrez” package was used to retrieve summaries of high-level evidence from PubMed; finally, the retrieved abstracts were re-submitted to the LLM, which generated evidence-based scores. In this evidence-based scoring prompt, the LLM was instructed to evaluate whether the extracted treatment was supported by high-level medical evidence, such as systematic reviews, meta-analyses, randomized controlled trials, or clinical guidelines. Treatments with stronger and more directly relevant evidence received higher scores, whereas treatments lacking reliable evidence or associated with unsupported claims received lower scores. The model was instructed to base its judgment on the retrieved PubMed evidence and to output both the score and the rationale. The complete prompt for abstract analysis is provided in Supplementary Appendix S3.

#### 2.3.2 Validity and reliability testing

To examine test-retest reliability, a simple random sample of 50 posts was drawn from the cleaned dataset. The same 50 posts were scored in two independent API runs using identical prompts, model version, and preprocessing procedures. Consistency between runs was evaluated using Spearman's rank correlation coefficient and the Intraclass Correlation Coefficient (ICC). For preliminary human validation, a stratified random sample of 20 posts was selected, with five posts from each of the four platform-source categories: Weibo professional-sourced, Weibo lay-sourced, REDnote professional-sourced, and REDnote lay-sourced posts. Two authors independently scored these posts according to predefined criteria and resolved disagreements through discussion to generate consensus manual scores. Paired-sample Wilcoxon signed-rank tests were used to compare LLM-based and consensus manual ratings, and agreement was summarized using Spearman's rank correlation coefficient, ICC, exact agreement, and mean absolute difference. Inter-rater reliability between the two human raters was not quantified before consensus; therefore, the manual comparison should be interpreted as validation against a consensus reference rather than as an independently established expert standard.

### 2.4 Statistical analyses

Appropriate statistical tests were used for group comparisons. When comparing linguistic feature scores across the four categories, data distribution did not meet normality assumptions; therefore, non-parametric Kruskal–Wallis tests were first applied to assess overall differences. Where significant, Dunn's *post-hoc* pairwise comparisons were performed, with Bonferroni correction applied to adjust *P*-values and control for Type I error due to multiple testing. For comparisons of categorical distributions of evidence-based content scores, Chi-square tests were used for overall differences, and Bonferroni correction was also applied to subsequent pairwise comparisons. All statistical analyses were conducted with the “rstatix” and “ggpubr” packages, and corrected *P*-values less than 0.05 were considered statistically significant.

## 3 Results

### 3.1 Descriptive analysis: information volume, user engagement, and content overlap

**Figure 1:**
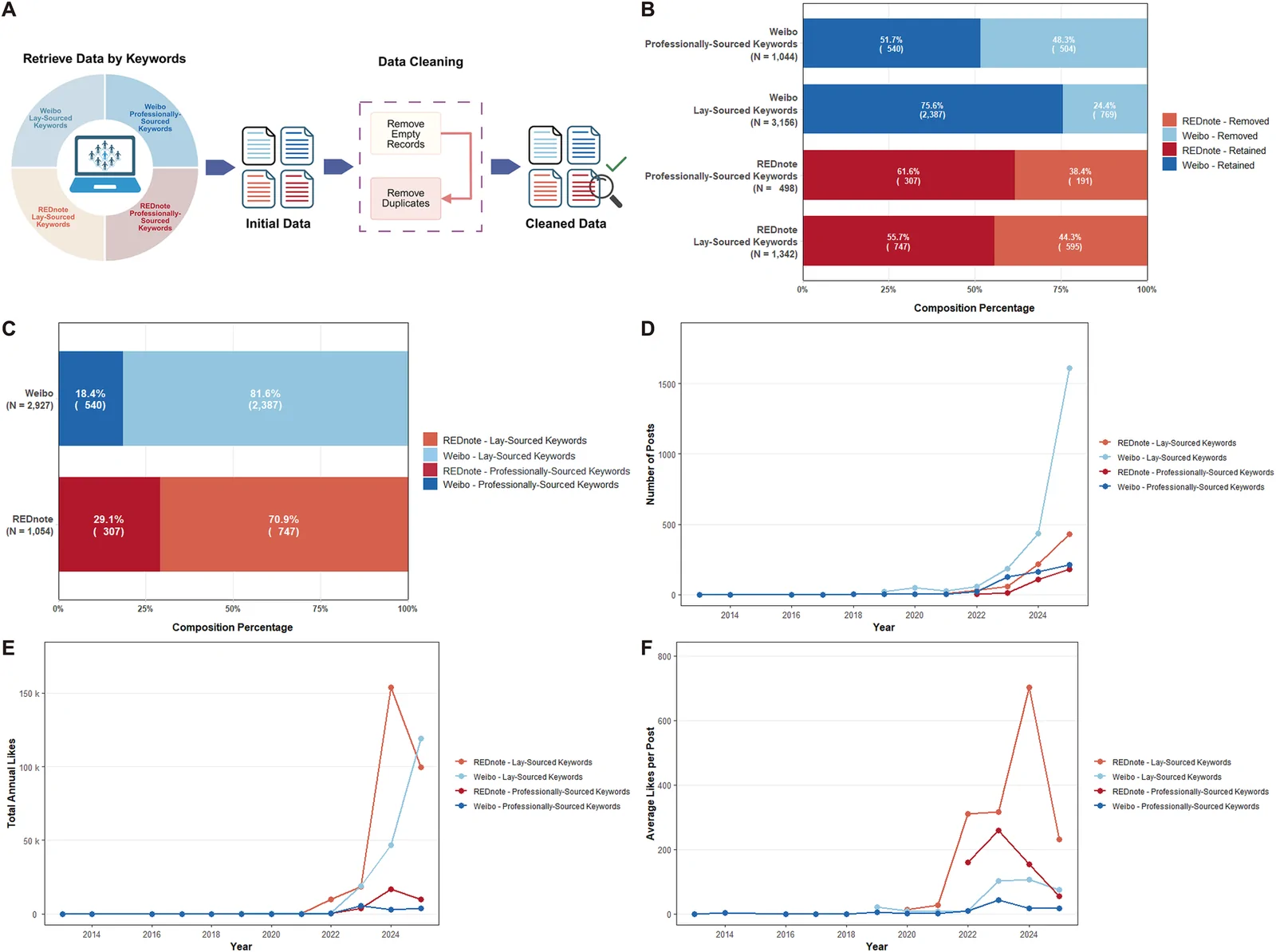
Descriptive analysis of information volume and user engagement across platforms and sources. **(A)** Flowchart of the data cleaning and inclusion process; **(B)** bar chart comparing the total number of posts on Weibo and REDnote before and after data cleaning; **(C)** proportional composition of posts retrieved using lay-sourced vs. professionally-sourced keywords on each platform; **(D)** line chart showing the annual trend of post volume for the four source categories from 2013 to 2025; **(E)** line chart showing the annual trend of total likes for the four source categories; **(F)** line chart showing the annual trend of average likes per post for the four source categories.

After cleaning the data collected from both platforms, a preliminary descriptive analysis was conducted (Figure 1A). The total volume of information on Weibo was consistently larger than that on REDnote, both before and after cleaning (Figure 1B). Across information sources, lay-sourced content dominated both platforms (Figure 1C). Among all categories, the Weibo-Lay group produced the highest volume of posts (*n* = 2,387), followed by REDnote-Lay with 747 posts; professional-sourced content was more limited, with 540 posts in Weibo-Pro and 307 in REDnote-Pro. These findings indicate that ordinary users contributed more IS treatment posts than professional institutions or practitioners in the analyzed dataset. From a temporal perspective, post volumes across all categories rose sharply after 2022–2023, with particularly steep growth in the lay-sourced categories (Figure 1D, Supplementary Material S1A). Because the analysis was descriptive, the factors underlying this temporal pattern cannot be determined from the present data.

In terms of user engagement, measured by the number of likes, lay-sourced content demonstrated overwhelming dominance (Figure 1E, Supplementary Material S1B). REDnote-Lay achieved approximately 281k likes, ranking first and reflecting strong user appeal. Weibo-Lay also performed well, with about 186k likes. By contrast, professional-sourced content attracted far less engagement: REDnote-Pro received about 30k likes, while Weibo-Pro obtained only 12k. Given the much larger volume of lay-sourced posts, average likes per post were further calculated to balance this discrepancy (Figure 1F, Supplementary Material S1C). On this metric, REDnote showed a distinct advantage. Lay-sourced content on REDnote achieved the highest average likes per post, peaking at 701.8 in 2024. Professional-sourced content ranked second, with an average of 259.1 likes in 2023. In comparison, Weibo-Lay peaked at 106.6 in 2024, and Weibo-Pro reached only 43.6 in 2023.

Overall, lay-sourced content dominated both quantity and engagement, particularly on REDnote. This descriptive association is compatible with greater engagement around widely disseminated or emotionally resonant content, but the study design cannot determine which content features caused user interaction.

To further explore the boundaries between information sources, a content overlap analysis was conducted. Results revealed a modest degree of overlap between professional and lay-sourced content within the same platform, but no overlap between platforms. Specifically, Weibo showed 25 overlapping posts between Pro and Lay categories, while REDnote showed 30 overlaps (Supplementary Figure S2). Given their small proportions (0.6% on Weibo and 0.8% on REDnote), these overlaps are unlikely to affect overall findings. Instead, they reflect the real-world blending of professional discourse and lay narratives. Accordingly, these posts were retained in subsequent analyses.

### 3.2 Regional distribution analysis

Geographic information provided by post authors was analyzed to describe regional variation in stroke-related content production and engagement. The results showed marked geographic differences in both measures.

**Figure 2:**
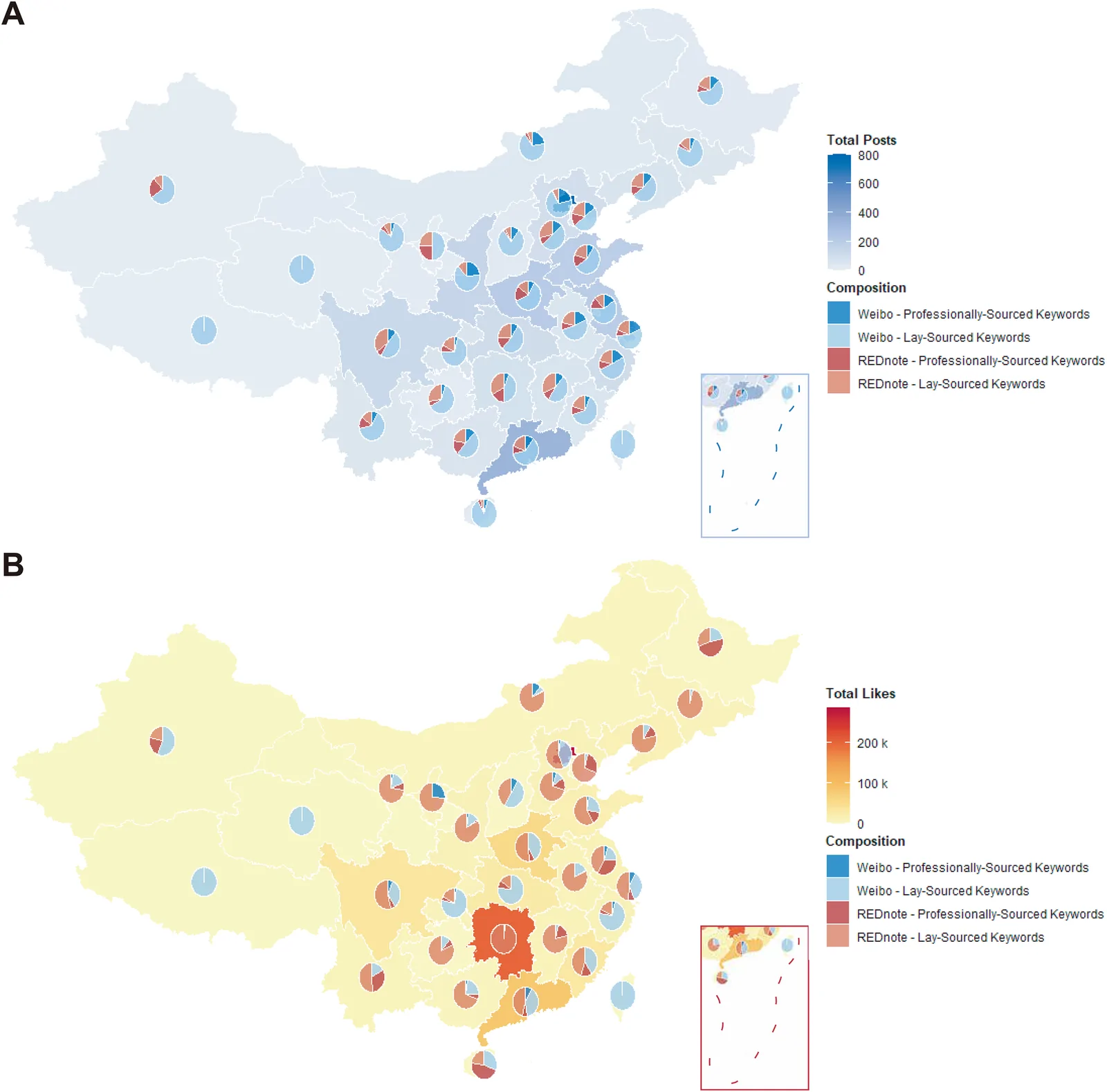
Geographic distribution of posts and user engagement across mainland China. **(A)** Choropleth map illustrating the provincial distribution of post volume. The background color of each province indicates the total number of posts, while the embedded pie chart shows the proportional composition of the four source categories; **(B)** choropleth map illustrating the provincial distribution of total likes. The background color indicates the total number of likes, and the pie chart shows its composition across the four source categories.

In terms of content production, posts were highly concentrated in China's political and economic centers. As shown in Table 1 and Figure 2A, Beijing led with 805 posts, followed by Guangdong (341 posts), Henan (212), Jiangsu (201), and Shandong (194). These regions also tend to have greater medical-resource availability and internet penetration, but the present analysis cannot determine whether those factors account for the observed concentration. By contrast, western and remote regions such as Qinghai, Ningxia, and Tibet contributed only single-digit posts. These low counts are consistent with, but do not by themselves establish, a digital divide in stroke health information dissemination.

**Table 1:** Number of posts by provinces.

Provinces	Weibo		REDnote		Total posts
	Lay-sourced keywords	Professionally-sourced keywords	Lay-sourced keywords	Professionally-sourced keywords	
Beijing	558	176	60	11	805
Guangdong	211	33	68	29	341
Henan	126	18	31	37	212
Jiangsu	122	29	25	25	201
Shandong	111	17	39	27	194
Shanghai	99	32	35	11	177
Shaanxi	95	36	19	1	151
Sichuan	60	14	44	8	126
Zhejiang	56	17	23	10	106
Hubei	55	10	26	14	105
Hebei	45	12	25	8	90
Hunan	34	5	26	13	78
Jiangxi	28	7	19	5	59
Liaoning	31	7	14	6	58
Guangxi	27	7	13	9	56
Chongqing	40	2	10	4	56
Anhui	29	10	12	4	55
Fujian	34	4	11	5	54
Tianjin	23	7	10	7	47
Jilin	33	3	6	2	44
Yunnan	28	3	6	6	43
Inner Mongolia	21	7	2	1	31
Shanxi	22	3	2	1	28
Heilongjiang	16	3	5	2	26
Hainan	21	1	1	1	24
Gansu	16	1	2	1	20
Guizhou	11	1	5	1	18
Xinjiang	6	0	1	2	9
Qinghai	4	0	0	0	4
Ningxia	2	0	1	1	4
Xizang	1	0	0	0	1
Taiwan	1	0	0	0	1

User engagement displayed a different geographic pattern. As shown in Table 2 and Figure 2B, Beijing ranked first in total likes (∼286k), consistent with its large content base. Hunan Province produced only 78 posts (ranked 12th nationwide) but accumulated about 200k likes, ranking second overall. Nearly all of Hunan's engagement came from REDnote-Lay posts (∼197k likes). This concentration was associated with a small number of REDnote-Lay “stroke recovery diary” posts; the descriptive analysis does not establish why these posts received high engagement.

**Table 2:** Number of likes by provinces.

Provinces	Weibo		REDnote		Total likes
	Lay-sourced keywords	Professionally-sourced keywords	Lay-sourced keywords	Professionally-sourced keywords	
Beijing	1,18,319	6,932	1,59,280	1,566	2,86,097
Hunan	1,452	43	1,96,783	2,318	2,00,596
Guangdong	34,177	6,781	39,454	4,372	84,784
Henan	23,397	392	28,623	3,483	55,895
Sichuan	11,306	2,415	18,821	2,136	34,678
Fujian	13,641	74	15,184	4,528	33,427
Hubei	21,349	14	4,127	2,568	28,058
Shanghai	7,874	1,742	10,566	1,732	21,914
Shandong	4,769	491	11,375	2,747	19,382
Liaoning	1,511	0	13,085	2,156	16,752
Jiangsu	2,737	770	5,998	4,628	14,133
Hebei	1,302	613	8,860	1,748	12,523
Zhejiang	9,234	498	2,173	597	12,502
Shaanxi	1,620	230	10,206	263	12,319
Jiangxi	476	48	8,743	1,924	11,191
Anhui	1,824	82	9,107	76	11,089
Chongqing	7,927	280	1,828	554	10,589
Jilin	332	0	8,907	50	9,289
Yunnan	1,419	1	4,565	2,886	8,871
Guangxi	1,833	159	5,474	468	7,934
Ningxia	0	1,872	5,378	10	7,260
Tianjin	197	17	3,659	1,421	5,294
Inner Mongolia	286	578	4,206	40	5,110
Heilongjiang	447	0	636	1,006	2,089
Guizhou	118	0	854	81	1,053
Hainan	267	0	197	394	858
Shanxi	390	70	334	2	796
Xinjiang	317	0	122	134	573
Taiwan	189	0	0	0	189
Gansu	28	2	115	13	158
Xizang	9	0	0	0	9
Qinghai	3	0	0	0	3

Together, the regional analysis documents two descriptive patterns: content production was concentrated in several eastern regions, whereas engagement included both concentrated activity and localized high-engagement clusters. These patterns should not be interpreted as evidence that regional resources or platform mechanisms caused the observed differences.

### 3.3 Content analysis

To systematically examine content differences across the four source categories, this study applied a two-stage topic modeling strategy. An initial LDA analysis identified broad thematic differences, which were then further characterized and refined using BERTopic.

#### 3.3.1 Exploratory LDA identifies broad thematic differences

**Figure 3:**
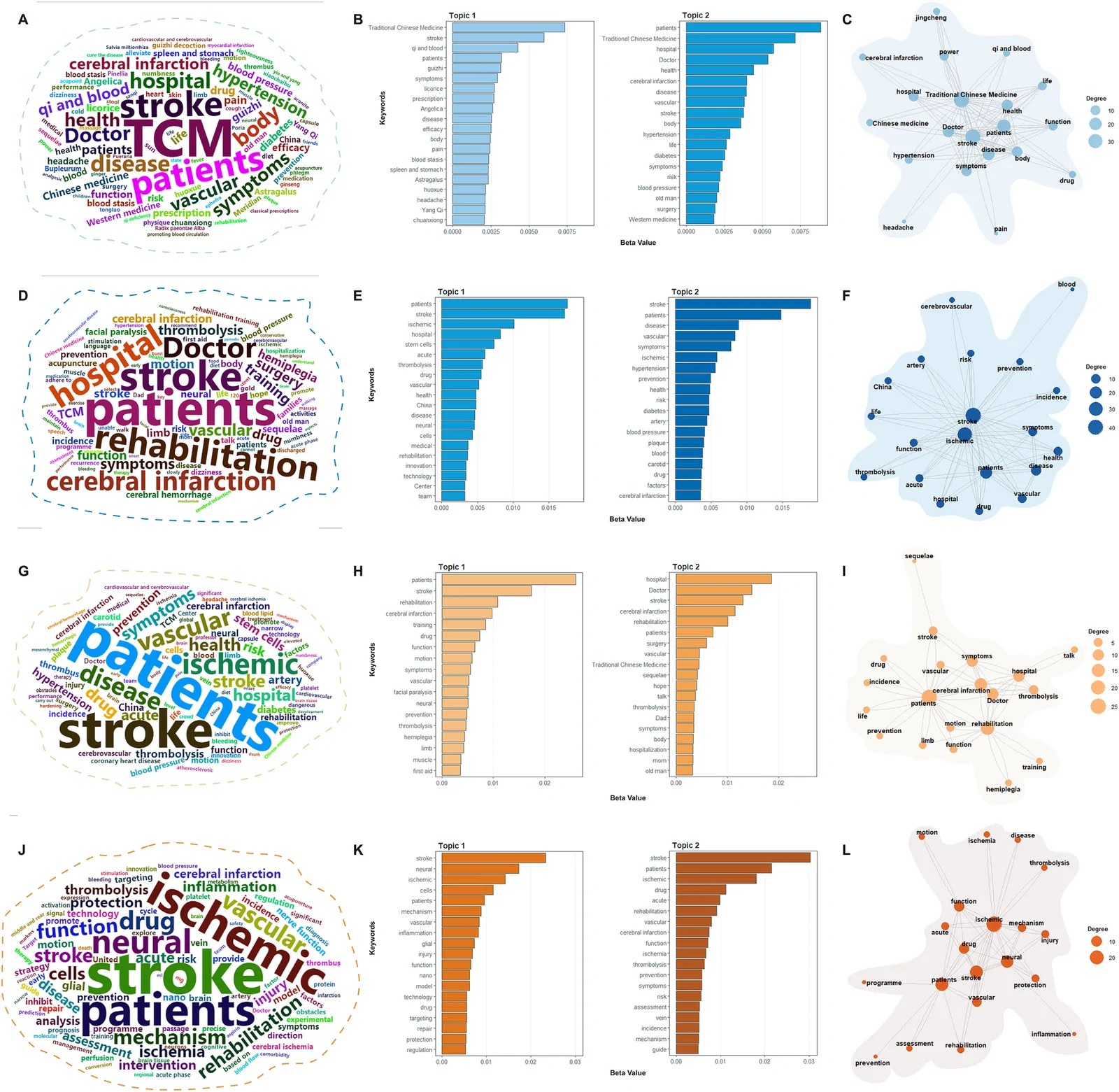
Comprehensive content analysis of the four source categories. This figure presents a multi-panel analysis for each of the four sources. For each source, the analysis includes: **(A,D,G,J)** Word clouds visualizing the top 100 most frequent keywords; **(B,E,H,K)** bar charts showing the top 20 keywords for each of the two topics identified by latent dirichlet allocation (LDA) topic modeling. The length of the bar represents the keyword's beta value (probability of the word belonging to the topic); **(C,F,I,L)** Keyword co-occurrence networks. Each node represents a keyword, and an edge represents the co-occurrence of two keywords within the same post. The size of the node is proportional to its degree centrality, indicating its importance in the network.

Lay-sourced content prominently reflected patient perspectives and personal experiences, with notable differences between platforms. In Weibo-Lay, “Traditional Chinese Medicine (TCM)” emerged as the top keyword, even surpassing “patient” and “stroke” (Figure 3A, Table 3). The LDA model identified a “TCM therapy” topic (keywords: “TCM”, “qi-blood”, “Guizhi”, “prescriptions”) alongside a “medical experiences and symptoms” topic (Figure 3B). Its co-occurrence network (Figure 3C) placed “TCM” as the central node linking “patient”, “disease”, and “health.” This pattern indicates that TCM terminology was prominent in Weibo-Lay discussions; it does not by itself establish demand for complementary and alternative therapies.

**Table 3:** Top 20 words in wordclouds.

Freq rank	Weibo		REDnote	
	Lay-sourced keywords	Professionally-sourced keywords	Lay-sourced keywords	Professionally-sourced keywords
1	Traditional Chinese Medicine	Patients	Patients	Stroke
2	Patients	Stroke	Stroke	Ischemic
3	Stroke	Ischemic	Rehabilitation	Patients
4	Disease	Disease	Hospital	Neural
5	Body	Vascular	Doctor	Drug
6	Hospital	Stroke	Cerebral infarction	Vascular
7	Doctor	Health	Vascular	Mechanism
8	Symptoms	Symptoms	Symptoms	Function
9	Vascular	Drug	Training	Stroke
10	Health	Hospital	Surgery	Rehabilitation
11	Hypertension	Acute	Thrombolysis	Cells
12	Cerebral infarction	Risk	Drug	Ischemia
13	Qi and blood	Stem cells	Motion	Acute
14	Patients	Prevention	Function	Protection
15	Guizhi	Artery	Stroke	Inflammation
16	Life	Hypertension	Traditional Chinese medicine	Disease
17	Pain	Thrombolysis	Hemiplegia	Thrombolysis
18	Drug	Diabetes	Cerebral infarction	Assessment
19	Chinese medicine	Cerebral infarction	Limb	Injury
20	Diabetes	China	Neural	Intervention

By contrast, REDnote-Lay centered on rehabilitation. “Rehabilitation” ranked third in frequency, just after “patient” and “stroke” (Figure 3G, Table 3). The LDA model identified two main themes: “post-surgical rehabilitation and functional training” (keywords: “rehabilitation”, “training”, “surgery”, “exercise”, “hemiplegia”, “limbs”) and “hospital experiences and emotional support” (keywords: “hospital”, “doctor”, “father”, “mother”, “hope”) (Figure 3H). In the co-occurrence network (Figure 3I), “rehabilitation” was a central node alongside “stroke” and “patient”, with links to “training”, “limbs”, and “function.” These patterns indicate that REDnote-Lay discourse was concentrated on recovery practices and family-centered narratives within a modern medical framework.

Professional-sourced content on both platforms was more medically oriented but showed distinct emphases. Weibo-Pro focused on clinical practice and public health education, with keywords such as “thrombolysis”, “prevention”, and “hypertension” (Figure 3D, Table 3). Its LDA topics were “acute treatment and technological innovation” (e.g., “thrombolysis”, “stem cells”) and “risk factor management and secondary prevention” (e.g., “hypertension”, “diabetes”, “risk”) (Figure 3E). The co-occurrence network (Figure 3F) was structured around “stroke” and “ischemic”, linked to terms such as “acute”, “thrombolysis”, and “risk”, a pattern consistent with a clinical popularization orientation.

REDnote-Pro contained more academic and mechanistic terminology. Alongside common terms (“stroke”, “patient”), frequent keywords included “neural”, “mechanism”, “function”, “cell”, and “inflammation” (Figure 3J, Table 3). Its LDA topics were “pathophysiology and cellular mechanisms” (e.g., “neural”, “cell”, “inflammation”, “protection”, “repair”) and “clinical interventions and rehabilitation guidelines” (e.g., “drugs”, “rehabilitation”, “guidelines”, “evaluation”) (Figure 3K). The co-occurrence network (Figure 3L) centered on “ischemic”, “neural”, and “mechanism”, with links to “injury”, “protection”, and “inflammation”, indicating an orientation toward basic science and disease mechanisms.

#### 3.3.2 In-depth BERTopic analysis further characterizes themes

The BERTopic analysis further characterized the broad thematic axes identified by LDA and provided a more granular view of the four source categories.

**Figure 4:**
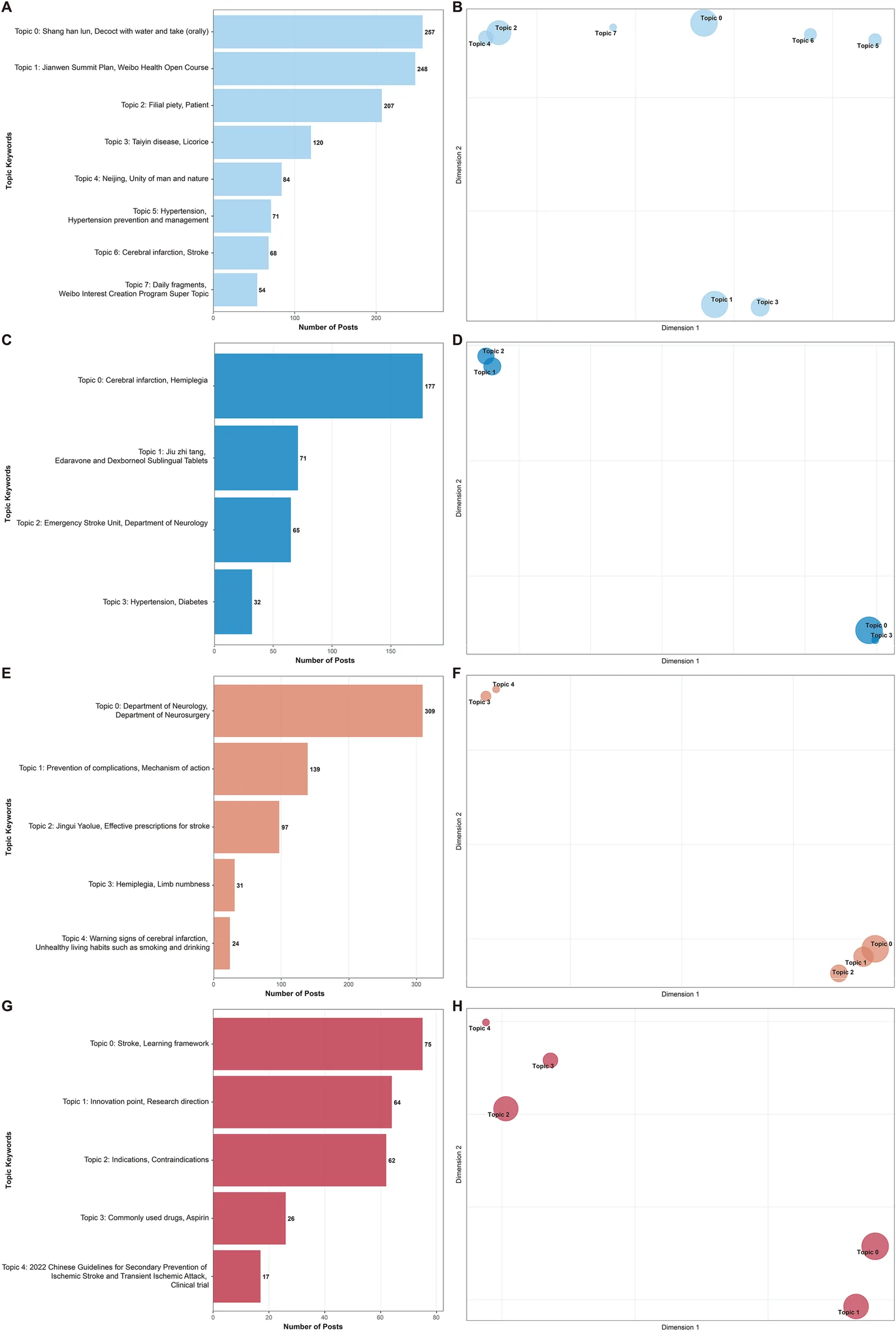
Thematic analysis and intertopic relationships using BERTopic across four source categories. This figure presents a comprehensive analysis of topics generated by BERTopic. The left panels **(A,C,E,G)** display horizontal bar charts of the most frequent topics identified within each source category. The length of each bar corresponds to the number of posts assigned to that topic, with labels indicating the top keywords. The right panels **(B,D,F,H)** show the two-dimensional projection of the intertopic distance map. In these maps, each circle represents a topic. The spatial distance between circles indicates the semantic similarity (closer circles are more similar), while the area of each circle is proportional to the topic's frequency. **(A,B)** Themes and their relationships from the Weibo-Lay source; **(C,D)** Themes and their relationships from the Weibo-Pro source; **(E,F)** Themes and their relationships from the REDnote-Lay source; **(G,H)** Themes and their relationships from the REDnote-Pro source.

Lay-sourced content emphasized patient perspectives and personal experiences, with different thematic structures across platforms. On Weibo-Lay, several high-frequency topics concerned classical TCM texts such as Shang Han Lun (Topic 0) and Nei Jing (Topic 4), as well as TCM doctors and herbal medicine principles (Topic 3) (Figures 4A,B). The intertopic distance map placed these topics in a cluster separate from more general health discussions. These patterns indicate a strong TCM-related thematic presence in Weibo-Lay posts. By contrast, REDnote-Lay topics were concentrated on contemporary healthcare navigation and personal experiences (Figures 4E,F). The most prominent topics included navigating clinical departments (“Department of Neurology, Department of Neurosurgery,” Topic 0), “Prevention of complications” (Topic 1), and “Warning signs” (Topic 4). A single classical text, Jingui Yaolue, was identified (Topic 2), but it was less prominent than topics related to contemporary hospital experiences. This pattern is consistent with a community discourse centered on practical, experience-based peer support.

Professional-sourced content was medically oriented on both platforms but differed in thematic emphasis. Weibo-Pro topics concerned acute-phase clinical practice and public health education (Figures 4C,D), including core clinical concepts (“Cerebral infarction, Hemiplegia,” Topic 0), emergency treatments (“Edaravone and Dexborneol Sublingual Tablets,” Topic 1), and hospital workflow (“Emergency Stroke Unit,” Topic 2). These themes were consistent with an emphasis on standard acute care and public education. REDnote-Pro contained more research-oriented topics (Figures 4G,H), including “Innovation point, research direction” (Topic 1), clinical-trial and guideline evidence (“2022 Chinese Guidelines,” Topic 4), and pharmacological interventions (“Commonly used drugs, Aspirin,” Topic 3). Together, the topic distributions indicate different professional communication emphases across the two platforms; they do not establish platform effects on professional behavior.

In sum, content analysis identified distinct thematic distributions: professional sources emphasized clinical education on Weibo and more mechanistic or research-oriented content on REDnote, whereas lay sources emphasized TCM on Weibo and rehabilitation plus family narratives on REDnote.

### 3.4 Analysis of viral content

Following the within-category operational definition, posts at or above the 75th percentile of likes were labeled “viral” (high-engagement), and a Structural Topic Model (STM) was used to compare topic prevalence between these posts and the remaining posts.

**Figure 5:**
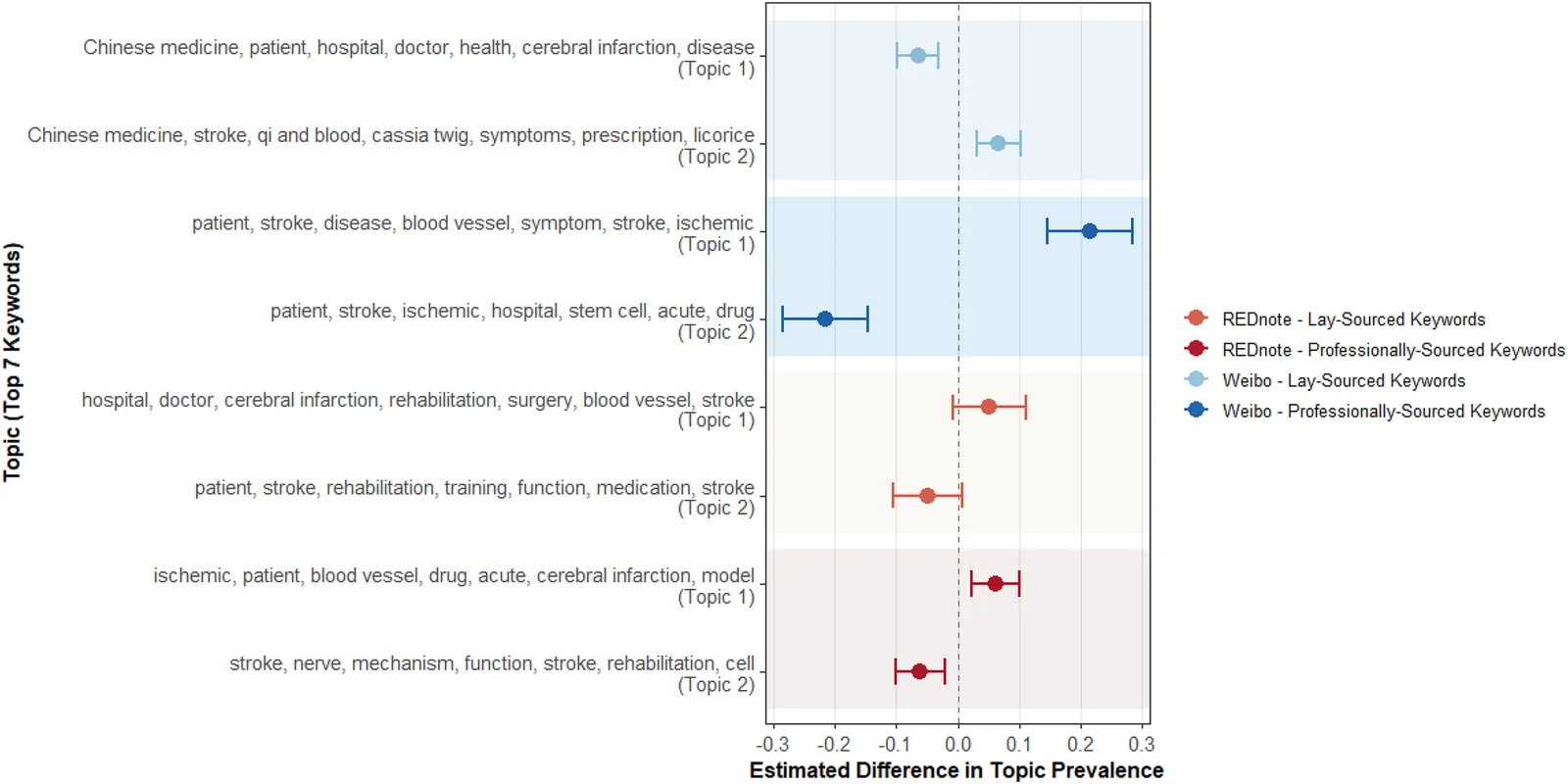
Forest plot of the structural topic model (STM) results, comparing topic prevalence in high-engagement vs. other content. Within each platform-source category, posts at or above the 75th percentile of likes were operationally classified as “viral” (high-engagement); this term denotes relative engagement within a category, not absolute virality. A positive estimate (to the right of the zero line) indicates that a topic is more prevalent in high-engagement content. Dots represent point estimates, and horizontal bars represent 95% confidence intervals. Each topic is labeled with its top seven keywords.

Results showed differences across platform-source categories (Figure 5). In Weibo-Lay, themes involving specific TCM prescriptions and theories (e.g., “TCM, stroke, qi-blood, Guizhi, prescriptions, licorice”) were more prevalent among high-engagement posts (estimate difference = 0.065), whereas general medical experiences were more prevalent among other posts. Thus, TCM prescription-related topics were associated with relatively higher engagement in Weibo-Lay; the analysis does not establish why these posts received more likes.

In Weibo-Pro, basic, accessible health-knowledge topics (keywords: “patient, stroke, disease, vessels, symptoms”) were more prevalent among high-engagement posts (estimate difference = 0.216), whereas advanced intervention topics (e.g., “stem cells, acute, drugs”) were less prevalent (estimate difference = –0.215). This indicates an association between accessible health-education topics and relatively higher engagement within Weibo-Pro, not a causal effect of topic type on dissemination.

On REDnote-Lay, complete personal medical narratives (e.g., “hospital, doctor, stroke, rehabilitation, surgery”) were slightly more prevalent among high-engagement posts (estimate difference = 0.051), whereas detailed rehabilitation topics (e.g., “rehabilitation, training, function, drugs”) were more prevalent among other posts. This pattern does not establish that storytelling or emotional resonance caused engagement.

In REDnote-Pro, practical clinical-knowledge topics (e.g., “ischemic, patient, vessels, drugs, acute”) were more prevalent among high-engagement posts (estimate difference = 0.061), whereas mechanistic research topics (e.g., “neural, mechanism, function, cell, rehabilitation”) were less prevalent. This association should not be interpreted as evidence of a general user preference or a causal dissemination mechanism.

Overall, high-engagement topic profiles differed by platform-source category: TCM prescription-related topics in Weibo-Lay, accessible education in Weibo-Pro, personal medical narratives in REDnote-Lay, and practical clinical information in REDnote-Pro were relatively more prevalent among posts in the upper quartile of likes.

### 3.5 Content quality scoring

**Figure 6:**
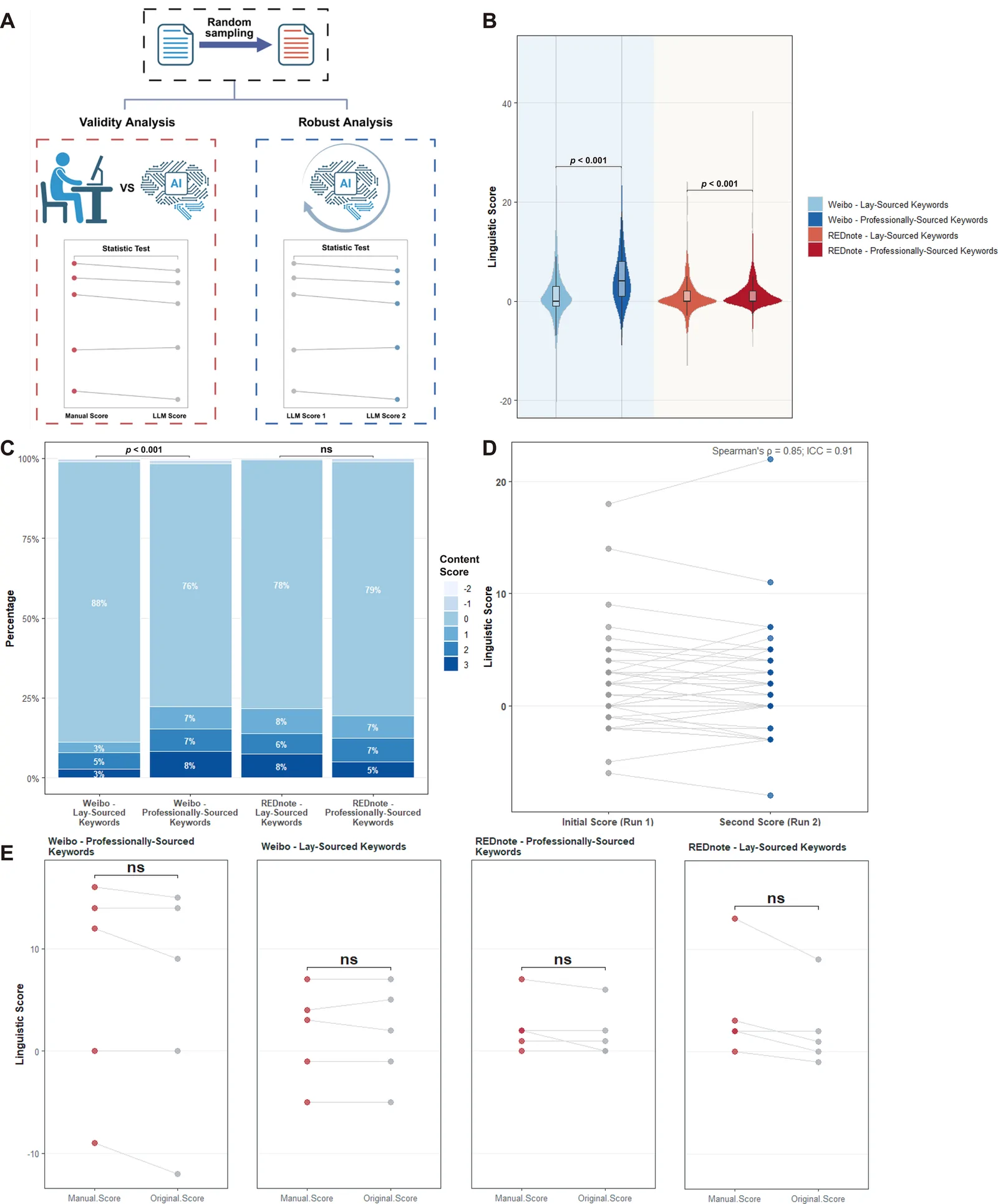
Content-quality scoring process and results. **(A)** Workflow for test-retest reliability and preliminary human validation of the LLM-based scoring system; **(B)** violin plots comparing linguistic-feature scores across the four source categories. Statistical significance was determined using the Kruskal–Wallis test followed by Dunn's *post-hoc* test with Bonferroni correction. ***p < 0.001; **(C)** stacked bar chart showing the distribution of evidence-based medical content scores (–3 to +3) across the four categories. Statistical significance was determined using the Chi-square test with Bonferroni correction for pairwise comparisons. ***p < 0.001; ns, not significant; **(D)** paired plot of two independent LLM runs on the same random sample of 50 posts using identical prompts, model version, and preprocessing procedures. Spearman's rank correlation coefficient (*ρ*) and the Intraclass Correlation Coefficient (ICC) are displayed; **(E)** paired plot comparing LLM-generated scores with consensus manual ratings in a stratified random sample of 20 posts. The consensus ratings were generated by two authors after discussion; human inter-rater reliability was not quantified before consensus. *P*-values from paired-sample Wilcoxon signed-rank tests are displayed. ns, not significant.

To quantitatively evaluate content quality, this study applied a two-dimensional LLM-based scoring system covering linguistic features and evidence-based medical content. Test-retest reliability and preliminary agreement with consensus manual ratings were examined (Figure 6A).

On linguistic features, professional-sourced content significantly outperformed lay-sourced content (*p* < 0.001, Figure 6B). Professional posts were more objective, accurate, logical, and standardized, while lay content tended toward colloquial, subjective, and emotional expression.

For evidence-based content, platform-level differences were observed (Figure 6C). On Weibo, professional-sourced content scored significantly higher than lay-sourced content (*p* < 0.001). In Weibo-Lay, 88% of posts scored 0 (no evidence-based content), compared with 76% in Weibo-Pro. On REDnote, no statistically significant difference was detected between professional and lay sources, and nearly 80% of posts in both categories scored 0. The absence of a significant difference on REDnote is a descriptive finding. It may reflect multiple factors, including presentation norms, the high proportion of zero scores in both groups, and the smaller REDnote-Pro sample; it does not demonstrate that REDnote culture reduced the salience of evidence.

Test-retest reliability was high between two independent LLM runs on the same 50 posts (Spearman's *ρ* = 0.85, ICC = 0.91; Figure 6D). Preliminary validity analysis found no significant differences between LLM and consensus manual ratings across the four categories (Figure 6E). Agreement between LLM-based scores and consensus manual scores was high by rank correlation and ICC (Spearman's *ρ* = 0.959, ICC = 0.968), while the exact score agreement rate was 45.0% (9/20) and the mean absolute difference was 1.05 points. Human inter-rater reliability was not quantified before consensus. These results support preliminary agreement with a consensus manual reference but do not establish that the automated system can substitute for manual evaluation. A summary of all four source categories is presented in Table 4.

**Table 4:** Comparative portrait of four digital health discourse communities.

Feature dimensions	Weibo		REDnote	
	Lay-sourced keywords	Professionally-sourced keywords	Lay-sourced keywords	Professionally-sourced keywords
Primary Platform Function	Public Square/Information Feed	Public Square/Information Feed	Interest Community/Lifestyle Hub	Interest Community/Lifestyle Hub
Dominant Content Theme	Traditional Chinese Medicine Therapies	Clinical Diagnosis, Treatment & Preventive Science Communication	Rehabilitation Training & Emotional Support	Academic Frontiers & Pathological Mechanisms
Core Knowledge Framework	Folk Wisdom/TCM Theories	Evidence-Based Medicine/Clinical Guidelines	Personal Experience/Peer Support	Basic Science/Research Progress
Key Interaction Driver	Practicality/Alternative Solutions	Authority/Timeliness	Empathy/Storytelling/Relatability	In-depth Explanation/Professionalism
Average Linguistic Feature Score	Low	High	Low	High
Evidence-Based Content Score	Extremely Low	Medium (Significantly higher than lay-sourced)	Medium	Medium (No significant difference from lay-sourced)

### 3.6 Proof-of-concept browser extension

**Figure 7:**
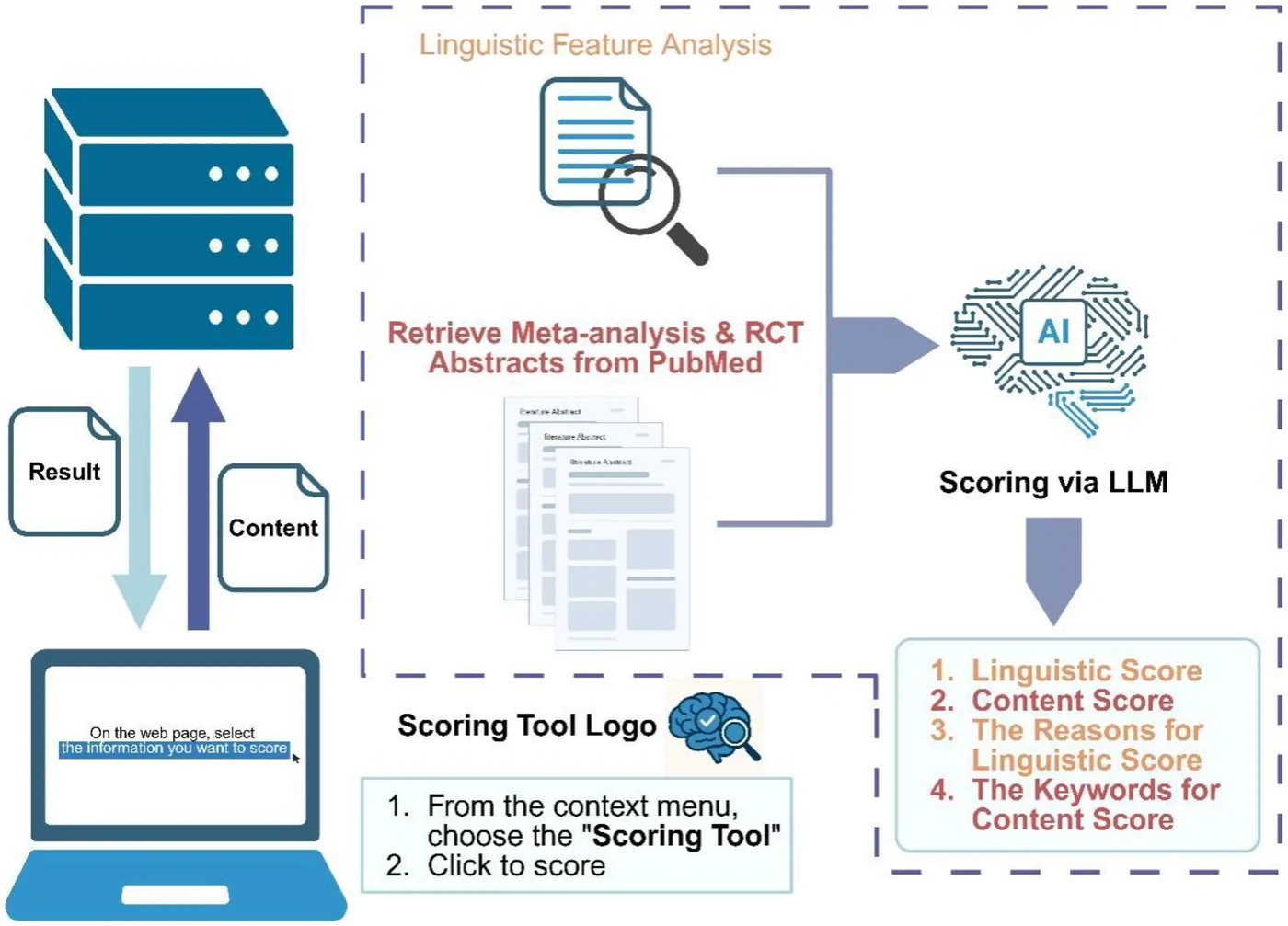
Interface of the proof-of-concept browser extension for health-information quality assessment. The screenshot illustrates a possible implementation in which selected webpage text is sent to the scoring modules and the generated scores and rationales are displayed. The prototype was not evaluated for usability, real-world accuracy, clinical utility, or effectiveness.

To illustrate one possible implementation of the scoring framework, a prototype browser extension was developed. When a user highlights text, the prototype sends it to the linguistic and evidence-based scoring modules and displays the generated scores and rationales. This section is descriptive only. The prototype was not evaluated for usability, accuracy in real-world browsing, user acceptance, clinical utility, or behavioral effects; therefore, no claims about practical applicability or effectiveness are made (Figure 7).

## 4 Discussion

This study systematically compared IS treatment information published by professional and lay sources on Weibo and REDnote. The analysis identified four distinct “discursive communities” associated with platform and source categories, with differences in content themes, user engagement, and information quality. Because the study was cross-sectional and observational, these patterns should be interpreted as associations rather than effects of platform culture.

Weibo is an open public forum with communication features closer to traditional mass media and an emphasis on timeliness and authority (14). In our sample, Weibo professional posts more often concerned clinical education and acute-phase treatment and had higher evidence-based scores than lay posts. REDnote is a lifestyle-sharing platform centered on visuals and personal narratives (15). In our sample, professional and lay posts on REDnote had similarly low evidence-based scores, a pattern we describe as a “quality paradox.” One possible interpretation is that platform-specific presentation norms are associated with convergence in how professional and lay content is expressed. However, the data cannot determine whether platform culture produced this pattern. The high proportion of zero scores in both REDnote groups, the smaller number of REDnote-Pro posts, residual differences in topic composition, and unmeasured account-level factors may also have contributed. The observed convergence should therefore be treated as hypothesis-generating rather than as evidence that REDnote eliminates source-based quality differences.

The predominance of lay content in quantity and engagement is consistent with prior accounts of social media enabling patients and caregivers to share experiences and peer support (16, 17). In this dataset, experience-based posts often received high engagement, but the analysis cannot determine whether emotional resonance or other content features caused that engagement. The high-engagement content analysis similarly showed associations between topic type and likes: REDnote rehabilitation narratives and Weibo TCM prescription-related topics were more prevalent among posts in the upper engagement quartile.

Furthermore, the findings of this study both align with and extend existing literature. First, the dominance of lay sources in online health discussions is consistent with research in other disease domains. Second, the observation that professional content demonstrates superior linguistic formality over lay content corroborates earlier studies (18). Therefore, these findings should not be interpreted as entirely novel in themselves. Rather, they provide contextual confirmation of previously observed patterns within the specific setting of IS treatment information on Chinese social media. However, the core contribution of this study lies in jointly examining platform type, information source, thematic structure, user engagement, and multidimensional quality scoring within the same analytical framework. While prior research has broadly emphasized the low quality of online health information (19), or simply contrasted professional vs. lay sources, this study suggests that platform context may be associated with variation in evidence-based quality patterns, particularly the apparent convergence between professional and lay content on REDnote. This finding should be interpreted cautiously, but it highlights the importance of considering platform-specific communication environments when evaluating online health information quality. In addition, the observed strong focus on TCM among Weibo lay users resonates with the broader sociocultural complexity of TCM in China, where its discussion is not only a health topic but also deeply tied to cultural identity and national positioning (20).

Methodologically, a strength of our study is the use of a complementary two-stage topic modeling strategy. Although the combined use of LDA and BERTopic is not entirely novel and has become increasingly common in computational social science, this approach was useful for balancing interpretability and contextual sensitivity in the present analysis. LDA provided an exploratory overview, whereas BERTopic offered a more context-aware characterization of subtopics. The broad agreement between the two approaches reduces, but does not eliminate, the possibility that the thematic patterns were artifacts of a single modeling method.

These findings may have preliminary implications for patients, healthcare professionals, and platform managers. For users, the results suggest that information source and platform context may be useful cues when cross-checking health content, but they do not establish that any specific strategy improves decisions. Clinicians may consider adapting presentation formats across platforms while preserving evidence-based content, although this approach requires empirical testing. Platform operators could investigate whether source labels, evidence indicators, or fact-checking prompts improve information quality or user judgment. Because no intervention, algorithmic change, or user outcome was evaluated, these proposals should be treated as directions for future testing rather than evidence-based recommendations.

Several limitations of this study must be acknowledged. First, although a comprehensive keyword library was constructed, some posts using unconventional or implicit expressions may have been missed. The cross-sectional design and fixed observation window also preclude analysis of dynamic changes and causal relationships. Second, test-retest consistency and preliminary agreement with consensus manual ratings do not establish the accuracy of LLM-based scoring or its equivalence to expert review. LLM-based scoring is also time-sensitive because model versions, API behavior, and underlying model updates can change, which may affect reproducibility. Third, the manual reference scores were produced by study authors; inter-rater reliability was not quantified before consensus, and no independent external raters were used. These features may introduce assessment bias and limit the strength of the validation. Fourth, the study focused on Chinese-language posts from Weibo and REDnote, so the findings may not generalize to other linguistic, cultural, regulatory, or platform settings. Finally, the study analyzed content and engagement rather than users' cognition, decisions, or health outcomes; those outcomes require evaluation through independent surveys, experiments, and field studies.

The browser extension was included only as an exploratory implementation example. It was not part of a comparative effectiveness analysis and should not be interpreted as a validated decision aid or intervention. Future studies would need to evaluate technical accuracy in live browsing, usability, user acceptance, potential harms, and effects on information judgment before practical claims can be made.

## 5 Conclusion

Through a multidimensional comparison of IS treatment information on Weibo and REDnote, this study identified platform- and source-related differences in content, engagement, and information quality. Weibo-Lay posts contained more TCM-related discourse, whereas REDnote-Lay posts emphasized rehabilitation and family narratives. Professional and lay posts on REDnote showed similar evidence-based score distributions in this sample, but the cross-sectional design, smaller REDnote-Pro sample, and other unmeasured factors preclude causal interpretation. The findings provide a platform-specific description of online IS treatment information and support further confirmatory and user-centered research. The automated scoring framework and browser extension should be regarded as preliminary research prototypes; their practical accuracy, usability, and effects require independent validation before use in public or clinical decision support.

## Data Availability

The original contributions presented in the study are included in the article/Supplementary Material, further inquiries can be directed to the corresponding author/s.
