# Supplementary material for: A comparative study on the credibility of ischemic stroke treatment information on social media platforms: evidence from Weibo and REDnote
Source: Front Digit Health. 2026 Aug 26;8:1858554. doi: 10.3389/fdgth.2026.1858554 (PMC13553954; doi:10.3389/fdgth.2026.1858554)
Supplement: Supplementary file 2 [file Datasheet2.docx]

**Supplementary Appendix S2: Prompt for Core Treatment/Keyword Extraction**

--- Instructions Begin ---

Please act as a rigorous medical information analyst.

Your task is to read the content below and identify the single most central therapy, drug, or substance being discussed.

Requirements:

Focus only on methods or substances directly used for treating or conditioning the body.

Ignore non-therapeutic terms such as people (e.g., 'father', 'TCM doctor'), places (e.g., 'hospital'), body parts (e.g., 'blood vessel'), and general concepts (e.g., 'health', 'qi-blood').

If the post does not contain a clear therapy, drug, or substance, or if it is primarily expressing emotions or narrating a story, please answer 'NA'.

Your answer must be very concise, containing only the name of that core therapy, without any additional explanation.

--- Post Content Below ---

[The content of the social media post is inserted here]

--- Your Answer (Please return only the core therapy or NA) ---
