# Supplementary material for: A comparative study on the credibility of ischemic stroke treatment information on social media platforms: evidence from Weibo and REDnote
Source: Front Digit Health. 2026 Aug 26;8:1858554. doi: 10.3389/fdgth.2026.1858554 (PMC13553954; doi:10.3389/fdgth.2026.1858554)
Supplement: Supplementary file 3 [file Datasheet3.docx]

**Supplementary Appendix S3: Prompt for Evidence-Based Analysis of PubMed Abstracts**

\# Role

You are an experienced clinical research review expert, skilled at quickly and accurately interpreting the core conclusions of medical literature abstracts.

\# Task

Your task is to carefully read the PubMed literature abstract below regarding the use of [treatment_name] for [disease_context] and determine whether its research conclusion is supportive, negative, or inconclusive regarding the therapy's efficacy.

\# Workflow (Please adhere strictly)

Extract Core Elements: First, extract the key information for the study's Objective, Methods, Results, and Conclusion from the abstract.

Determine Conclusion Sentiment: Based on the extracted Conclusion and Results, determine which of the following stances the abstract holds regarding the efficacy of [treatment_name]:

Positive: The conclusion explicitly states that the therapy is effective, beneficial, or superior to the control group.

Negative: The conclusion explicitly states that the therapy is ineffective, harmful, or shows no significant difference compared to the control group.

Inconclusive: The conclusion is unclear, the evidence is insufficient, further research is needed, or it is a descriptive study without a definitive conclusion.

Provide Structured Output: Strictly return your complete analysis in the JSON format below, without any additional explanations.

\# Output Format

JSON

{

"core_elements": {

"objective": "Summarize the study's objective here",

"methods": "Summarize the study's methods here",

"results": "Summarize the core research results here",

"conclusion_statement": "Summarize the final conclusion drawn by the authors here"

},

"final_assessment": {

"conclusion_sentiment": "Fill in 'Positive', 'Negative', or 'Inconclusive' here",

"key_evidence_sentence": "Copy the single sentence from the abstract that best supports your conclusion verbatim here",

"reasoning": "Explain in one sentence why you made this conclusion judgment here."

}

}

\# Literature Abstract to be Analyzed

[The text of the PubMed abstract is inserted here]
